# 同时性多原发肺腺癌的外科治疗及预后

**DOI:** 10.3779/j.issn.1009-3419.2017.02.05

**Published:** 2017-02-20

**Authors:** 岳 彭, 晖 王, 厚耐 谢, 万刚 任, 振 冯, 猛 李, 忠民 彭

**Affiliations:** 250021 济南，山东大学附属省立医院胸外科 Department of Thoracic Surgery, Shandong Provincial Hospital Affiliated to Shandong University, Jinan 250021, China

**Keywords:** 肺肿瘤, 表皮生长因子受体, 预后, Lung neoplasms, Epidermal growth factor receptor, Prognosis

## Abstract

**背景与目的:**

随着高分辨率计算机断层扫描（high-resolution computed tomography, HRCT）应用的普及，多原发肺癌（multiple primary lung cancers, MPLC）的检出率逐年上升，其中腺癌是最常见的病理类型。目前国内外对MPLC的研究已相对多见，但罕有单独分析同时性多原发肺腺癌（synchronous multiple primary lung adenocarcinomas, SMPLA）的报道。本研究探讨SMPLA患者的临床病理特点及预后，旨在提高对SMPLA的认识。

**方法:**

对2012年12月-2016年7月期间我科38例临床资料保存完整的SMPLA患者进行了回顾性分析。

**结果:**

38例SMPLA患者中，男性12例，女性26例，中位年龄为58岁（39岁-73岁）。双原发肺腺癌29例，2个病灶以上9例。病灶位于同侧26例，双侧12例。同期手术34例（包括8例患者同期行双侧手术），分期手术4例。5例患者对每个病灶分别行基因检测，结果证实不同病灶的表皮生长因子受体（epidermal growth factor receptor, *EGFR*）基因突变类型不完全相同。1年和3年总生存率分别为96.6%和74.2%。肿瘤直径越大（*P* < 0.001）、T分期越高（*P*=0.003）、淋巴结转移（*P*=0.001）、TNM分期越高（*P*=0.022）以及术后放、化疗（*P*=0.009）提示总生存预后较差。

**结论:**

对于多发的非小细胞肺癌，不能轻易地诊断为转移癌，应考虑多原发可能。*EGFR*基因检测可作为鉴别多原发肺腺癌与复发转移癌的临床参考。

## Abstract (en)

**Background and objective:**

With the popularization of high-resolution computed tomography (HRCT), there is a rising trend of the detection of multiple primary lung cancers (MPLC). Adenocarcinomas is the major pathological type of MPLC. At present, reports on MPLC are relatively common, but few study focused on synchronous multiple primary lung adenocarcinomas (SMPLA). We carried out this study in an attempt to enhance our understanding about SMPLA.

**Methods:**

Data from 38 patients undergoing surgery for SMPLA in our institution frrom December 2012 and July 2016 were retrospectively collected.

**Results:**

Among the 38 patients, 12 patients were male, 26 patients were female, with a median age of 58 (ranging from 39 to 73). Surgical outcomes verified 29 patients with 2 tumors and 9 patients with more than 2 tumors. There were 26 patients with tumors in ipsilateral lung while 12 patients in contralateral lung. Eight patients underwent one-stage surgical treatment for contralateral tumors with mean postoperative hospitalization of 10 days. The gene detection results of 5 patients showed different epidermal growth factor receptor (*EGFR*) mutations can be found in one patient's different tumors. The 1-year and 3-year overall survival (OS) rate were 96.6% and 74.2%. Larger maximal tumor dimension (*P* < 0.001), advanced pT stage (*P*=0.003), lymph nodes metastases positive (*P*=0.001), advanced TNM stage (*P*=0.022) and postoperative adjuvant chemoradiotherapy (*P*=0.009) were correlated with poor OS.

**Conclusion:**

Multiple lung malignant lesions should not be taken as metastasis for granted and the possibility of MPLC also should be considered. Mutational status of *EGFR* could be used as a clinical reference to diagnose patients with SMPLA.

多原发肺癌（multiple primary lung cancers, MPLC）是指同一个患者一侧或双侧肺内同时或先后发生两个或两个以上组织学类型相同或不同的原发性恶性肿瘤，可以分为同时性多原发肺癌（synchronous multiple primary lung cancers, SMPLC）和异时性多原发肺癌（metachronous multiple primary lung cancers, MMPLC）。随着现代诊断技术如高分辨率计算机断层扫描（high-resolution computed tomography, HRCT）的进步，MPLC的检出率逐步上升，既往研究中高达8.0%经外科手术治疗的非小细胞肺癌患者术后被证实为MPLC，其中腺癌是最常见的病理类型^[1-4]^。

最早在1924年，Beyreuther首次发现并报道了MPLC，而后在1975年，Martini和Melamed^[5, 6]^首次确立和发表了MPLC的诊断标准，该诊断标准可以辅助临床鉴别MPLC和肺内转移癌。2003年，美国胸科医师协会（American College of Chest Physicians, ACCP）对Martini-Melamed标准进行了修订和补充，引入分子基因诊断作为标准，利用不同分子遗传学特点鉴别相同病理的不同病灶^[7]^。然而，从MPLC被发现和报道以来的92年中，国内外尚无任何机构制订发表权威和系统的治疗指南。

既往国内外对MPLC的研究中，较少有单独分析SMPLA的报道，本研究回顾性分析了我院胸外科2012年11月-2016年7月的38例同时性多原发肺腺癌（synchronous multiple primary lung adenocarcinomas, SMPLA）患者的治疗及预后，旨在提高对SMPLA的认识。

## 1 材料和方法

### 1.1 患者资料

本研究对2012年12月-2016年7月我院胸外科38例临床资料保存完整的SMPLA患者的外科治疗和预后进行回顾性分析。研究中SMPLA患者的诊断参照Martini-Melamed^[6]^诊断标准并结合2013年ACCP指南的改良诊断标准^[8]^：①不同病灶肺腺癌的亚型不同；②不同病灶分子基因学特征不同；③肿瘤位于不同肺段或肺叶，起源于不同部位的原位癌灶，无肺癌共同淋巴引流部位及肺外转移。

### 1.2 术前评估和手术方案

患者术前常规行胸部正侧位片、胸部强化CT、颅脑磁共振成像（magnetic resonance imaging, MRI）或CT、全身骨扫描、腹部超声、心脏超声和肺功能检查。针对SMPLA外科治疗的基本策略：①最大程度的切除肿瘤和最大程度的保留健康肺组织；②优先切除主要病灶[中央型肺癌病灶或肿瘤-淋巴结-转移（tumor-node-metastasis, TNM）分期最高的病灶]；③所有病灶位于同一侧肺的患者优先考虑同期手术；④病灶位于双侧肺的患者根据患者对手术耐受程度考虑行同期或分期手术治疗。手术方式包括肺叶切除和亚肺叶切除（肺段切除和楔形切除）。

### 1.3 病理分期和术后随访

患者术后病理分期按照第8版肺癌TNM分期对每个病灶进行单独分期^[9]^，并采取最高分期作为患者的最终分期。患者术后前2年每3个月复查1次；术后第3到5年，每6个月复查1次；术后5年以后，每年复查1次。术后复查检查包括胸部CT、肿瘤标志物、腹部超声，根据患者情况可以考虑复查颅脑MRI、全身骨显像。

### 1.4 数据分析

总生存期（overall survival, OS）定义为从首次手术日期到死亡日期或末次随访日期；无复发生存期（disease free survival, DFS）定义为从首次手术日期到复发转移日期或末次随访日期。本研究最终随访时间点为2016年11月15日。

### 1.5 统计学方法

采用SPSS 22.0统计学软件对数据进行分析，采用*Kaplan*-*Meier*法绘制无复发生存曲线和总生存曲线，并用*Log*-*rank*检验比较各组生存曲线间的差异。采用*Cox*风险比例回归模型进行多因素分析。*P* < 0.05为差异有统计学意义。

## 2 结果

### 2.1 临床病理学特征

38例SMPLA患者中，男性12例，女性26例，年龄39岁-73岁，中位年龄为58岁。有吸烟史的患者8例，其余30例患者无吸烟史。有家族恶性肿瘤史2例（表 1）。

**1:** 临床病理学特征与无病生存期/总生存期之间的关系 The relationship between clinicopathologic features and DFS/OS

Characteristics	*n* (%)	*P*	
		DFS	OS
Age (yr)		0.494	0.529
< 60	22 (57.9)		
≥60	16 (42.1)		
Gender		0.835	0.829
Male	12 (31.6)		
Female	26 (68.4)		
Smoking history		0.501	0.701
Yes	8 (21.1)		
No	30 (78.9)		
Family history of cancer		0.682	0.270
Yes	2 (5.3)		
No	36 (94.7)		
Number of tumors		0.457	0.965
2	29 (76.3)		
≥3	9 (23.7)		
Largest tumor dimension (cm)		< 0.001	< 0.001
1 cm < d≤2 cm	19 (50.0)		
2 cm < d≤3 cm	13 (34.2)		
3 cm < d≤4 cm	5 (13.2)		
4 cm < d≤5 cm	1 (2.6)		
Tumor location		0.624	0.476
Ipsilateral lung	26 (68.4)		
Same lobe	10		
Different lobes	16		
Contralateral lung	12 (31.6)		
Lymph node metastases		0.046	0.001
Yes	6 (15.8)		
No	32 (84.2)		
pT stage		< 0.001	0.003
1b	15 (39.5)		
1c	6 (15.8)		
2a	16 (42.1)		
2b	1 (2.6)		
pTNM stage		0.082	0.022
Ia2	14 (36.8)		
Ia3	6 (15.8)		
Ⅰb	12 (31.6)		
Ⅱb	1 (0.03)		
Ⅲa	5 (13.2)		
Adjuvant chemoradiotherapy		< 0.001	0.009
Yes	14 (36.8)		
No	24 (63.2)		
DFS: disease free survival; OS: overall survival; pTNM: pathological tumor-node-metastasis.			

### 2.2 临床治疗

手术治疗情况见表 2。38例SMPLA患者均通过手术切除所有病灶，均未发生围手术期死亡。其中29例行胸腔镜手术，8例行开胸手术，余1例患者行开胸联合胸腔镜同期切除双侧病灶（左肺下叶切除、胸腔镜右肺上叶楔形切除、淋巴结清扫术）。分期手术4例。同期手术34例包括8例患者行同期双侧开胸手术，术后平均住院日为10 d（8 d-12 d），其他单侧手术患者术后平均住院日为8.5 d（3 d-14 d）。12例患者行肺叶切除术，21例患者行肺叶切除联合亚肺叶切除术，5例患者行亚肺叶切除术。术后行放、化疗治疗患者共有14例。

**2:** 手术方式和病理结果 Surgical procedures and postoperative pathology

Characteristics	*n* (%)
Surgical stage	
Ipsilateral tumors	26
Single-stage	26 (100.0)
Contralateral tumors	12
Single-stage	8 (66.7)
Two-stage	4 (33.3)
Surgical approach	
Ipsilateral tumors	26
Thoracotomy	8 (30.8)
VATS	18 (69.2)
Contralateral tumors	12
VATS	11 (91.7)
Thoracotomy+VATS	1 (8.3)
Surgical resection type	
Lobectomy	12 (31.6)
Lobectomy+sublobectomy	21 (55.3)
Sublobectomy	5 (13.2)
Sum of tumors	94
ADC in situ	20 (21.3)
Minimally invasive ADC	4 (4.3)
Invasive ADC	70 (74.5)
VATS: video-assisted thoracoscopic surgery; ADC: adenocarcinoma.	

### 2.3 肿瘤特点和病理结果

肿瘤特点和病理结果见表 1和表 2。38例SMPLA患者中共切除94个腺癌病灶，其中双原发肺腺癌29例，其余9例患者病灶数量超过2个。病灶位于同侧26例（包括10例患者位于同一肺叶），病灶位于双侧12例。术后病理示浸润性腺癌病灶70个，微浸润性腺癌病灶4个，原位腺癌病灶20个。所有患者中主病灶直径平均为2.3 cm（1.3 cm-5 cm），1例存在N1淋巴结转移，5例患者存在N2淋巴结转移。根据第8版TNM分期，Ia2期14例，Ia3期6例，Ⅰb期12例，Ⅱb期1例，Ⅲa期5例。22例术后行基因检测，其中17例术后仅检测主病灶，5例对每个病灶分别行基因检测，结果证实不同的病灶的基因突变型不同（表 3）。

**3:** 5例患者对不同病灶分别行基因检测的结果 Gene detection result in 5 patients for different lesions respectively

	Lesion 1	Lesion 2	Lesion 3	Lesion 4
Case 1	L858R	Wild	-	-
Case 2	L858R	Wild	L858R	Wild
Case 3	19-Del	Wild	-	-
Case 4	G719X	L858R	-	-
Case 5	L858R	Wild	Wild	Wild

### 2.4 数据分析

38例患者按照第8版TNM分期的术后总生存曲线列于图 1中。术后中位随访期为24.2个月（4.4个月-47.9个月），随访率94.7%。1年和3年总生存率分别为96.6%和74.2%，1年和3年的无病生存率分别为93.6%和71.8%。单因素分析结果显示肿瘤直径越大（*P* < 0.001）、T分期越高（*P*=0.003）、淋巴结转移（*P*=0.001）、TNM分期越高（*P*=0.022）以及术后放化疗（*P*=0.009）与较差的OS有关。肿瘤直径越大（*P* < 0.001）、T分期越高（*P* < 0.001）、淋巴结转移（*P*=0.046）以及术后放化疗（*P* < 0.001）与较差的DFS有关（表 1）。多因素分析未得出有统计学意义的结果。

**1:**
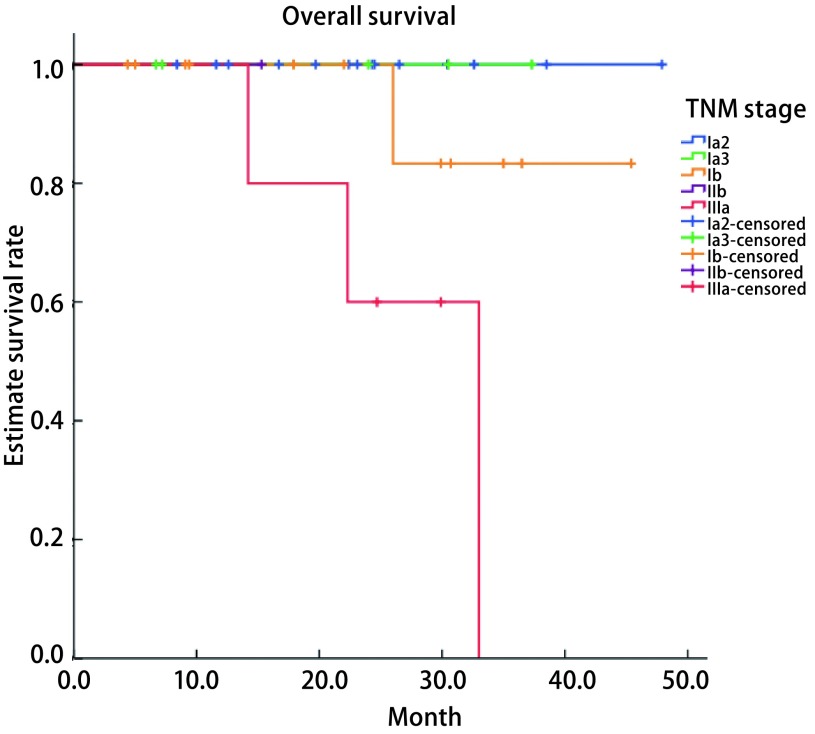
38例SMPLA患者总生存函数（*P*=0.022） Overall survival of the 38 SMPLA patients (*P*=0.022). SMPLA: synchronous multiple primary lung adenocarcinomas.

## 3 讨论

第7版和第8版TNM分期标准均指出同一肺叶的不同病灶应考虑为T3期，同侧肺不同肺叶的病灶应考虑为T4期，而双侧肺叶的病灶应考虑为M1a期^[9, 10]^。这种分期方式导致了许多MPLC患者被过度分期为Ⅱ期/Ⅲ期/Ⅳ期，不少患者因此丧失了手术治疗的机会，而只能接受放化疗、靶向治疗、免疫治疗等保守治疗。MPLC应根据不同的病灶分别分期，采取最高分期作为患者的最终分期，并基于最高分期制定治疗方案。但当前的书写模式并不能充分显示整体病灶分期情况，对此我们建议：对病灶较少的患者可在T分期及N分期后分别同时标注，两病灶间用顿号分开，例如：同侧3个病灶分别为4 cm、1 cm、0.8 cm，无肺门及纵隔淋巴结转移，则为T_2_、_1_、_1_N_0_M_0_；不同侧标注左右，如左T_1_、_1_、_1_N_1_M_0_，右T_2_、_1_N_0_M_0_。

美国国立综合癌症网络（National Comprehensive Cancer Network, NCCN）非小细胞肺癌治疗指南（2017，第2版）指出，临床诊断为MPLC的患者，如果存在病变进展到出现临床症状的高危因素、淋巴结转移分期考虑为N0期或者N1期且无胸外转移，若局部治疗可行应首选手术治疗^[11]^。在以往的对MPLC的研究中，采取外科手术治疗的3年总生存率为40.0%-92.0%^[12-14]^，5年总生存率为34% -77.6%^[12-18]^，这明显较指南中复发和转移癌的5年生存率高：Ⅱb期为53%；Ⅲa期为36%；Ⅲb期为26%；Ⅲc期为13%；Ⅳa期为10%^[11]^。同时，既往的一些研究证实MPLA患者在MPLC患者中所占的比例最高，且MPLA的外科治疗预后大多优于混合型MPLC及多原发肺鳞癌等其他病理类型，这可能与纳入研究中的MPLA患者存在原位癌和微浸润癌病灶有关^[17, 19, 20]^。

MPLC的治疗方案的制定应基于其病灶的最高分期，尽管目前没有任何机构出版系统和权威的MPLC治疗指南，但对于早期MPLC应优先选择手术治疗逐渐得到了越来越广泛的认同。病灶位于不同肺叶或肺段的肺部多发病灶患者，若怀疑恶性可能性大，且胸部CT无纵隔淋巴结转移，应考虑积极行手术治疗，对无法完全外科切除或高度怀疑为肺内转移癌的患者进行放、化疗等内科治疗。手术治疗应遵循最大程度地切除肿瘤以及最小程度的切除健康肺组织。Zhang等^[17]^的治疗经验提倡避免同期双侧手术治疗MPLC，但我们的手术经验证实同期双侧手术是可行的。本研究中有12例同时性双侧多原发肺腺癌患者，其中8例行同期双侧开胸手术，术后平均住院日为10 d（8 d-12 d），其他单侧手术患者术后平均住院日为8.5 d（3 d-14 d），两组数据之间的差异无统计学意义。以我们的经验，对于心肺功能较好的患者可以考虑同期行双侧手术治疗。

本研究同先前的一些研究一致，肿瘤直径越大、T分期越晚、淋巴结转移以及术后化疗与较差的预后有关^[14, 17]^，而年龄、性别、吸烟史、肿瘤数量和分布、家族史都与预后无关，但Zhang等^[17]^的研究证实有恶性肿瘤家族史预后较好。这些不同的结论可能与样本量的差异有关。需要指出的是，术后辅助化疗的效果在目前仍有争议，但这个争议多针对于分期较早的MPLC患者。在既往表明术后辅助放、化疗有利于预后的研究中，多数是存在淋巴结转移的患者^[16, 18]^，而本研究中术后放、化疗与预后较差相关，分析原因是多数患者分期较早，淋巴结转移率很低，而术后接受放、化疗的患者分期较晚，这部分分期较晚接受术后放、化疗的患者与分期较早未接受放、化疗的患者相比预后较差。其他一些研究^[2, 14, 17, 19]^报告表明术后放、化疗和预后并没有关系，但这些报告中同样缺乏更为系统的分组对照研究，我们认为针对术后辅助治疗仍需进行更广泛和深入的临床研究。

MPLC的发病理论基础可以追溯到1953年，Slaughter等^[21]^通过研究口腔多原发鳞癌首次提出“区域癌化”的概念，认为口腔粘膜发生多原发癌是因为不同的癌前病变病灶，在相同的致癌因素（如吸烟等）作用下相互独立发生癌变，后来该理论逐渐延伸到呼吸道以及消化道多原发肿瘤。Strong等^[22, 23]^认为致癌因素长期作用于呼吸道粘膜，会导致广泛的细胞DNA损伤及异型增生，经过长期一系列基因突变的积累，不同位置的高级别异型增生细胞相继癌变，最终发展成独立的不同克隆来源的MPLC。由于此类癌变是一系列基因突变长期积累的最终结果，理论上讲，不同的多原发癌灶具有完全相同的基因表型的概率是几乎不存在的^[24, 25]^。而复发和转移性恶性肿瘤与原发肿瘤的遗传学特征相似，基因检测结果理应相同，这是分子基因检测用于鉴别诊断MPLC的理论依据。本研究中5例患者针对不同病灶分别进行基因检测，结果证实了同一个患者不同病灶存在不同的基因突变。Yatabe等^[26, 27]^对82例存在*EGFR*基因突变的肺腺癌患者的同一病灶进行多层切片、多点取材，重复检测*EGFR*基因突变的结果一致。另有77例患者分别对原发癌和转移癌的病灶和54例患者分别对原发癌和复发癌的病灶进行*EGFR*基因突变检测，结果也一致。

然而，其他一些研究表明，由于在转移癌和原发癌之间存在分子异质性，导致原发灶与转移灶之间存在不同的基因表型，这就使得MPLC与复发转移癌的鉴别诊断更为困难。相关研究证实原发灶与相应转移灶之间*EGFR*基因突变状况的不一致率在27%-33%之间，而MPLC病灶具有超过50%的不同的分子克隆^[28-30]^。目前临床上常用的基因检测为针对一些特定靶点检测的二代测序，ACCP认为这些手段仅能作为参考^[8]^。针对MPLC与转移癌的鉴别仍需要更深入和广泛的研究全基因组测序。

最后，本研究仍存在一些局限性。①样本量太少，同时由于本研究是单中心的回顾性研究会存在一定的选择偏倚；②基因检测未在所有患者中针对不同病灶进行分别检测；③本研究中多原发肺腺癌的手术患者未和单纯行保守治疗的患者进行对比研究。

对于多发的非小细胞肺癌，不能轻易地诊断为转移癌，应考虑多原发可能。*EGFR*基因检测可作为鉴别多原发肺腺癌与复发转移癌的临床参考。
